# Natural Products as an Emerging Therapeutic Alternative in the Treatment of Neurological Disorders

**DOI:** 10.1155/2018/3056847

**Published:** 2018-04-03

**Authors:** Nasiara Karim, Heba Abdelhalim, Navnath Gavande, Imran Khan, Haroon Khan

**Affiliations:** ^1^Department of Pharmacy, University of Malakand, Khyber Pakhtunkhwa, Pakistan; ^2^Faculty of Pharmacy and Medical Sciences, University of Petra, Amman, Jordan; ^3^School of Medicine, Indiana University, Bloomington, IN, USA; ^4^Department of Pharmacy, University of Swabi, Khyber Pakhtunkhwa, Pakistan; ^5^Department of Pharmacy, Abdul Wali Khan University, Mardan, Khyber Pakhtunkhwa, Pakistan

Neurological disorders are common and represent a major public health problem. Neurological disorders include dementia, epilepsy, headache disorders, multiple sclerosis, and neuroinfections, neurological disorders associated with malnutrition, pain associated with neurological disorders, Parkinson's disease, stroke, and traumatic brain injuries. There are approximately 450 million of world population suffering from these mental disorders [1]. For example, 50 million people have epilepsy and this number is increasing day by day [2]. The number of people suffering from dementia and memory disorders is projected to be doubled every 20 years. Currently, 322 million people suffer from major depression [3] and this number is on the rise. Neurological disorders constitute over 6% of the global burden of disease [4]. This burden is especially high in many low- and middle-income countries.

Considerable efforts have been made in recent decades to discover substances which can help prevent these serious neurological disorders. Natural products are small molecules found in divergent natural sources. They possess a coveted position in the treatment of all human illnesses including neurological disorders. They are believed to be the single most important source of drug leads [5]. The importance of plant derived natural products for the treatment of neurological disorders is evident by the fact that most of the earlier drugs used for the treatment of neurological disorders were derived from plants including opioids alkaloids [6], galantamine [7], and anticholinesterases like physostigmine and neostigmine [8]. During the last decade out of total 26 natural drugs approved, 7 were for the treatment of neurological disorders, out of which 3 were for Parkinson's disease [9].

This special issue focuses on original contributions for natural products being useful in various neurological disorders including anxiety, depression, stroke, epilepsy, and other CNS disorders and their possible mechanisms of action.
